# Influence of the Preparation Method and Photo-Oxidation Treatment on the Thermal and Gas Transport Properties of Dense Films Based on a Poly(ether-block-amide) Copolymer

**DOI:** 10.3390/ma11081326

**Published:** 2018-07-31

**Authors:** Gabriele Clarizia, Paola Bernardo, Giuliana Gorrasi, Daniela Zampino, Sabrina C. Carroccio

**Affiliations:** 1 Istituto per la Tecnologia delle Membrane, Consiglio Nazionale delle Ricerche (ITM-CNR), via P. Bucci 17/c, 87036 Rende, Italy; g.clarizia@itm.cnr.it; 2 Dipartimento di Ingegneria Industriale, Università degli Studi di Salerno, Via Giovanni Paolo II 132, 84084 Fisciano, Italy; ggorrasi@unisa.it; 3 Istituto per i Polimeri Compositi e Biomateriali, Consiglio Nazionale delle Ricerche (IPCB-CNR), via P. Gaifami 18, 95126 Catania, Italy; sabrinacarola.carroccio@cnr.it

**Keywords:** poly(ether-block-amide) copolymer, photo-oxidation, gas transport properties

## Abstract

Dense films based on the hydrophobic Pebax^®^2533 were prepared by using solution casting in different solvents as well as compression molding and subjected to photo–aging under ultraviolet (UV) irradiation. The influence of the preparation method, including the casting solvents, as well as the UV irradiation time selected to treat the samples, were evaluated in terms of permeation rates of pure gases (CO_2_, N_2_, O_2_, CH_4_, He, and H_2_). The transport data were correlated with the microstructure and surface properties by using differential scanning calorimetry (DSC), thermogravimetric analysis (TGA), matrix-assisted laser desorption ionization time-of-flight mass spectrometry (MALDI-TOF MS), as well as water contact angle measurements. The obtained results showed that a controlled photo-oxidation process reduces the hydrophobicity of the Pebax^®^2533 films, increasing their permeability without compromising their integrity.

## 1. Introduction

Pebax^®^, commercial elastomeric poly (ether-block-amide) copolymers, have interesting properties for textile, medical, and packaging applications as well as for membrane applications. Membrane gas separation processes are energy-efficient and low-cost solutions for a range of technologically relevant applications, such as hydrogen recovery, air separation, recovery of volatile organics from gas streams, and carbon dioxide capture [1].

Pebax^®^ are phase-separated block copolymers, consisting of polyether (PE) amorphous rubbery blocks and hard polyamide (PA) semi-crystalline segments. The soft PE blocks, owing to their high chain mobility, are gas permeable, while the hard PA segments provide mechanical stability. Different properties can be accomplished by changing the type of the PE and PA segments and their relative ratio [2,3]. Pebax grades with a higher fraction of the flexible PE segment are more hydrophobic. Pebax^®^2533 has the highest content of PE segment (84 wt.% of poly (tetramethylene oxide)) and a good gas permeability with respect to Pebax^®^1657 that contains a greater amount of the hard polyamide (60 wt.%). Therefore, according to the trade-off between selectivity and gas permeability for polymeric membranes, Pebax^®^2533 is more permeable, while Pebax^®^1657 is more selective [4]. Different studies have focused on Pebax^®^ as a membrane material [5,6,7] showing interesting results for the CO_2_/N_2_ separation, which is relevant to the CO_2_ capture from flue gas for greenhouse gas emission control. Sorption and permeation tests suggested strong interactions between the polar gas CO_2_ and the PE blocks in these copolymers [5]. In order to tailor the properties of Pebax^®^ membranes, different additives were considered, including ionic liquids [4,8] or nanoparticles [9,10]. Barbi et al. [3], studying different grades of Pebax^®^ dissolved in high boiling point alcohols (cyclohexanol or 1-butanol), showed that the gas permeability depends on the casting solvent.

The photo-oxidative resistance of the material is required for some applications (e.g., medical and packaging). Furthermore, in the case of membrane technology, the photo-oxidation process can be used as a valuable strategy to tune the separation performance. As far as this point is concerned, the understanding of the photo-oxidation and stability of polymeric membranes could be a crucial point to address towards their application for gas separation, catalysis, or sensors [11].

In the present study, Pebax^®^2533 was used for preparing dense films, investigating the effect of the casting solvent on their microstructure and transport properties. The range of the solvents investigated was extended to different alcohols. The alcohols comprise 1-butanol, but also solvents with a lower boiling point in order to facilitate their removal from the produced films. In addition, we take into account their eco-compatibility, as in the case of ethanol [12], and also their affinity with the polymer, as in the case of the fluorinated alcohol hexafluoroisopropanol (HFIP). HFIP has the same structure as isopropyl alcohol, but contains six fluorine atoms and, therefore, can make hydrogen bonds. Films obtained in a solvent-free procedure by melting the polymer pellets in a hot press were prepared as another green alternative and compared with the previous ones. Furthermore, film samples were subjected to a photo-oxidative process under UV irradiation up to 11 h. The oxidation level was monitored by using matrix-assisted laser desorption ionization time-of-flight mass spectrometry (MALDI TOF MS) analysis. The films’ gas transport properties were evaluated and correlated with microstructure and surface properties. Data obtained from photo-irradiated polymers were compared with the "as prepared” samples and the results herein reported.

## 2. Materials and Methods

### 2.1. Materials

**Figure 1:**
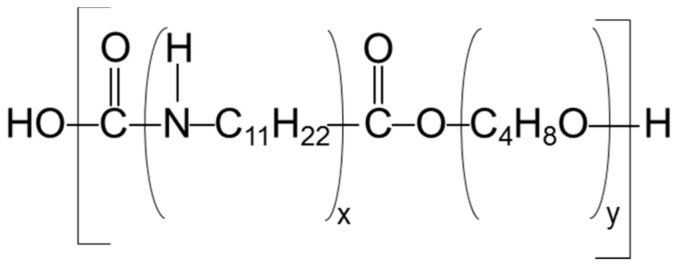
Chemical structure of Pebax^®^2533.

Pebax^®^2533 grade (Figure 1), a block copolymer of poly (tetramethylene oxide) (PTMO, 84 wt.%) and polyamide (PA12, 12 wt.%), was received in pellets from Arkema, Rho, Italy.

Ethanol (absolute, VWR), *i*-propanol (99.5%, Carlo Erba Reagenti), 1-butanol (99.5%, Carlo Erba Reagenti), and 1,1,1,3,3,3-hexafluoro-2-propanol (HFIP, Sigma-Aldrich) were used as solvents without further purification to dissolve the polymer. The solvents used, with some physical properties, are listed in Table 1. The 1,8-dihydroxy-9-anthrone (dithranol) was purchased from Sigma-Aldrich. The gases for the permeation tests (nitrogen, oxygen, methane, helium, hydrogen, and carbon dioxide, all with purity of 99.99+%) were supplied by SIAD, Bergamo, Italy.

### 2.2. Methods

#### 2.2.1. Membrane Preparation

The dissolution of Pebax^®^2533 pellets in ethanol, *i*-propanol, or 1-butanol required the heating under reflux conditions for ca. 2 h.

The membranes were prepared as dense flat sheets by controlled solvent evaporation, casting the dope solution within a stainless steel ring placed onto a Teflon support. The rings were covered and left at room conditions overnight. The films, having a thickness of ca. 150 micron, were then heated at 50 °C for a few hours.

In the case of HFIP, Pebax^®^2533 pellets were dissolved in the solvent under vigorous stirring at 50 °C for 1 h. Prefixed aliquots of mixture were cast on glass plates, followed by solvent evaporation overnight at room temperature to produce films ca. 150 ± 10 µm thick.

Solvent-free films with a thickness of 150 ± 10 µm were obtained by compression molding in a Carver laboratory press between two Teflon sheets, at 200 °C, followed by cooling at ambient temperature. No further thermal treatment was carried out on the molded samples.

#### 2.2.2. Membrane Post-Treatment: Photo-Oxidation

Photo-oxidative degradation of Pebax^®^2533 films was carried out on a QUV PANEL apparatus at 60 °C with continued exposure to UV radiation for up to 11 h in absence of water. At least two separate films were analyzed at each exposure time. The irradiance (0.68 W m^−2^) of the UV lamps (UVA 340 lamps) has a broad band with a maximum at 340 nm.

#### 2.2.3. Membrane Characterization

##### Matrix-Assisted Laser Desorption Ionization Time-of-Flight Mass Spectrometry (MALDI-TOF MS)

A 4800 MALDI TOF/TOFTM Analyzer (Applied Biosystems, Framingham, MA, USA) mass spectrometer was used in this study to acquire MALDI spectra. The TOF/TOF instrument is equipped with a (neodymium-yttrium-aluminum-garnet) Nd:YAG laser (355 nm wavelength) of <500 ps pulse and 200 Hz repetition rate. MALDI-TOF/TOF-MS spectra were recorded in reflector positive ion mode. The mass resolution of the MALDI spectra was about 10,000 (full width at half maximum, FWHM) and the mass accuracy was 1–10 ppm for masses in the range *m*/*z* 200–1000 Da. The grid voltage and the delay time were optimized for each sample to achieve the higher molar mass values and the best resolution. The laser irradiance was maintained slightly above threshold. Dithranol (0.1 M in HFIP) was used as matrix. Appropriate volumes of polymer solution (5 mg mL^−1^ in HFIP) and matrix solution were mixed to obtain 2:1, 1:1, and 1:2 ratios (sample/matrix *v*/*v*). The cationizing agent sodium trifluoroacetate (NaTFA) was used to improve the quality of spectra as well as to discriminate possible structures during the peak assignment. Then, 1 µL of each sample/matrix mixture was spotted on the MALDI sample holder and slowly dried to allow matrix crystallization. At least three separate portions were analyzed at each heating time. The resolution of the MALDI spectra reported in the text is about 9000 FWHM. The structural assignment of MALDI peaks in Table 2 was mainly made on the basis of empirical formulas. However, isotopic resolution helps considerably in the peak-assignment process through the comparison of the relative intensities of isotopic peaks corresponding to oligomers of increasing molar mass. When necessary, to distinguish and separate between the contribution of isotopic peaks M + 1 and M + 2 and peaks due to isobaric structures, a deisotoping program (Data explorer^®^ software, Applied Biosystems, Framingham, MA, USA) was used. The relative amount of the peak at *m*/*z* 1154.22 was obtained as the ratio IA/IB where IA is the peak intensity of the selected species and IB corresponds to the intensity of a base peak appearing in the selected mass range.

##### Contact Angle

Wettability measurements were carried out by using a DATAPHYSICS OCA 15 PRO apparatus and water as liquid. Direct measurements of the contact angles were performed on four different areas of the same sample. All measurements were carried out at room temperature.

##### Thermogravimetric Analysis (TGA)

TGA of the films submitted to photo-oxidation was performed using a Q500 thermogravimetric apparatus (TA Instruments—Division of Waters S.p.A., Sesto San Giovanni (MI), Italy) under air atmosphere (flow rate: 60 mL min^−1^) at 10 °C min^−1^ heating rate, from 40 °C to 600 °C. For comparison, unexposed films were analyzed too. The weight loss percent and its derivate (DTG) were recorded as a function of temperature.

##### Differential Scanning Calorimetry (DSC)

The thermal properties of the films were determined by a DSC 822 instrument (Mettler-Toledo S.p.A., Novate Milanese, Italy) under nitrogen atmosphere. The investigated temperature range was from −40 to 150 °C and the heating rate was 10 °C min^−1^.

#### 2.2.4. Gas Permeation Tests

The films were characterized via gas permeation tests conducted in constant volume/variable pressure equipment (Elektro & Elektronik Service Reuter, Geesthacht, Germany). Single gas permeability of H_2_, He, N_2_, O_2_, CH_4_, and CO_2_ was determined within the temperature range from 15 to 55 °C. The feed pressure was 1 bar. The instrument is equipped with a turbo molecular pump to evacuate the membrane samples before each experiment. The membrane was exposed to the feed gas, and the pressure increase in a calibrated volume at the permeate side was monitored by a pressure transducer. The increase of the pressure on the permeate side over time approaches the steady state asymptote indicated as a straight line whose slope yields the permeability (*P*):

(1) $$p_{t}=p_{0}+{\left(\frac{dp}{dt}\right)}_{t=0}\cdot \;t+\;\frac{R\;T\;A}{V_{P}\;V_{m}}\;\cdot \;\frac{p^{Feed}\;P}{l}\cdot \;\left(t-\theta \right)$$

where *p_t_* is the permeate pressure at time *t*, *p*_0_ the starting pressure, (*dp/dt*)_0_ the baseline slope, *R* the universal gas constant, *T* the absolute temperature, *A* the exposed membrane area, *V_P_* the permeate volume, *V_m_* the molar volume of the permeating gas at standard temperature and pressure (0 °C and 1 atm), *p_f_* the feed pressure, *l* the membrane thickness and *θ* the gas time-lag. Typically, the term *p*_0_ + *(dp/dt)*_0_ is negligible for defect-free samples that are well evacuated.

The extrapolation of the linear portion of the pressure vs. time curve provides the gas time-lag, *θ*. This parameter allows the evaluation of the diffusion coefficient, *D*, of each gas through the membrane [13]:

(2) $$D=\frac{l^{2}}{6\theta }$$

According to the ‘solution-diffusion’ transport mechanism in dense polymeric films [14], the solubility coefficient, *S*, was indirectly obtained as:

*S* = *P*/*D* (3)

The ideal selectivity (α_A/B_) is calculated as the ratio of the individual permeability values for two gases and can be decoupled into solubility-selectivity and diffusivity-selectivity:

*α*_A/B_ = *P*_A_/*P*_B_ = *S*_A_/*S*_B_ × *D*_A_/*D*_B_ (4)

The samples were circular, with an effective membrane area of 11.3 or 2.14 cm^2^. Their thickness was measured using a digital micrometer (IP65, Mitutoyo, Lainate (MI), Italy) and obtained as an average of at least five measurements.

## 3. Results

The Pebax^®^ films submitted to photo-oxidation were characterized by different techniques. The samples treated for a period of 11 h became fragile and thus could not be tested. For comparison, unexposed films were analyzed as well.

### 3.1. Surface Properties (MALDI MS and Water Contact Angle)

To evaluate changes in molecular architecture during the photo-oxidation, all virgin and photo-exposed Pebax^®^ samples were analyzed by MALDI MS. As well stated in the literature, the polymer degradation mainly yielded products bearing characteristic end groups, which can be revealed and differentiated by MS, being indicative of specific degradation pathways [15]. The identification of the end groups attached to the oligomers produced is of remarkable importance, since it may reveal the particular mechanism that has been active in the degradation processes. The oligomer structures assigned to the mass ions appearing in the MALDI spectra of virgin and photo-oxidized Pebax^®^ samples in the mass range 918–2618 Da are reported in Table 2.

**Figure 2:**
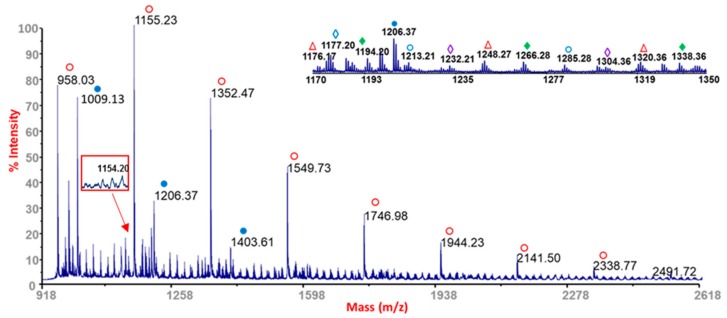
MALDI spectrum of virgin (T0) Pebax^®^ sample obtained from a hot press molded sample.

The comparison of the registered MALDI spectra of virgin Pebax^®^ samples prepared by solvent casting did not reveal significant changes. The MALDI spectra of the virgin Pebax^®^ samples showed the appearance of PA12 cyclic oligomers (*m*/*z* 1009.13 + *n* × 197.18) as well as PA12 linear chains having carboxylic terminal groups (*m*/*z* 1155.23 + *n* × 197.18). As it is possible to appreciate from the inset in Figure 2, at lower intensity, poly(tetramethylene oxide) cyclic (*m*/*z* 1176.17 + *n* × 72.1; 1232.21 + *n* × 72.1), and linear oligomers (*m*/*z* 1194.20 + *n* × 72.1) are also present. These species are most likely due to the unreacted co-oligomers or degradation products due to processing operations. In contrast, in the MALDI spectrum of the sample prepared by hot press, the peaks belonging to linear chains having carboxylic terminal groups (*m*/*z* 1155.23 + *n* × 197.18) appeared at higher intensities if compared with the cyclic ones.

**Figure 3:**
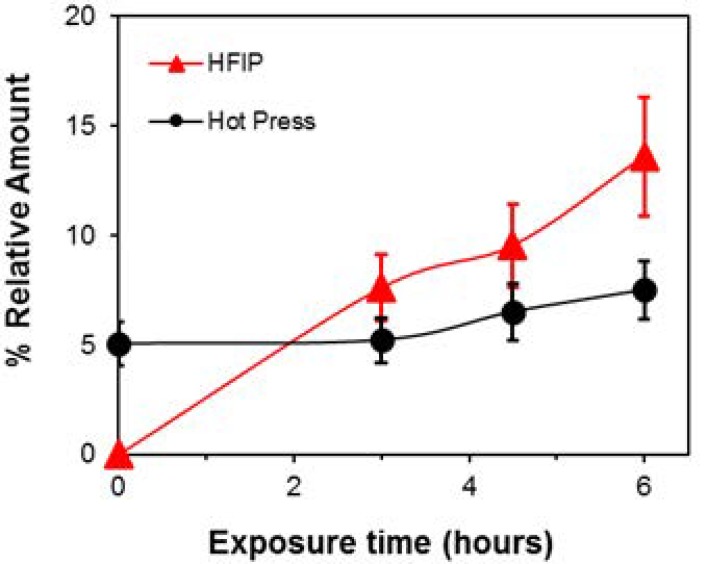
Relative amount (%) of a characteristic peak (*m*/*z* 1154.20) derived from the photoreaction process as a function of time exposure in a film cast from HFIP and in a hot press molded sample.

After UV exposure, in all samples, a peak at *m*/*z* 1154.22 occurred. However, as evidenced in Figure 2 and Figure 3, this peak is already present in the MALDI spectra collected from hot press samples at T0, whereas it was not detected at T0 in the samples obtained from the solvent casting technique. The presence of this species can be ascribed to a specific degradation oligomer formed during processing [16], having the same structure of a characteristic photoreaction product [17,18]. In the latter case, its intensity increased during the exposure time, confirming that photo-degradation is active, producing new oxidized species [19] onto the exposed Pebax surfaces. In contrast, this behavior was not very evident in the hot press samples after UV exposure, where its increase versus exposure time is low. This discrepancy can be ascribed to the initial presence of a thermo-degradation product that might affect the growth rate of the corresponding photo-degradation product detected at *m*/*z* 1154.22.

The static water contact angles of the unmodified and modified Pebax membranes were measured to evaluate changes induced on the film surface by the photo-oxidative treatment. Table 3 shows the effects of the preparation method and of the casting solvent on the surface properties of the ‘as prepared’ films. The water contact angle for the film cast in ethanol is comparable to that typically reported for PTFE [19]. The samples cast from ethanol or 1-butanol presented a more pronounced hydrophobic character with respect to those prepared with *i*-propanol or HFIP. Instead, a slightly hydrophilic surface was evidenced for the molded films, where the surface contact with the hot press can induce the formation of thermo-oxidized hydrophilic species.

As evidenced in Table 3, in the case of ethanol and HFIP, the water contact angle of the modified membranes tended to decrease with the treatment time because of the growth of hydrophilic domains induced by the photo-oxidation (see MALDI data, Figure 2 and Figure 3). Correspondingly, the same films showed larger gains in gas permeability after the photo-oxidative treatment, as will be discussed in the following. Nevertheless, minor changes upon the photo-oxidation can be observed for the films cast from *i*-propanol or 1-butanol and hot press samples. Films produced by the latter method showed the lowest water contact angle owing to the surface degradation occurring during their preparation. These data are in agreement with the higher carboxylic chain ends content found by the MALDI analysis.

### 3.2. Thermal Properties (TGA and DSC)

**Figure 4:**
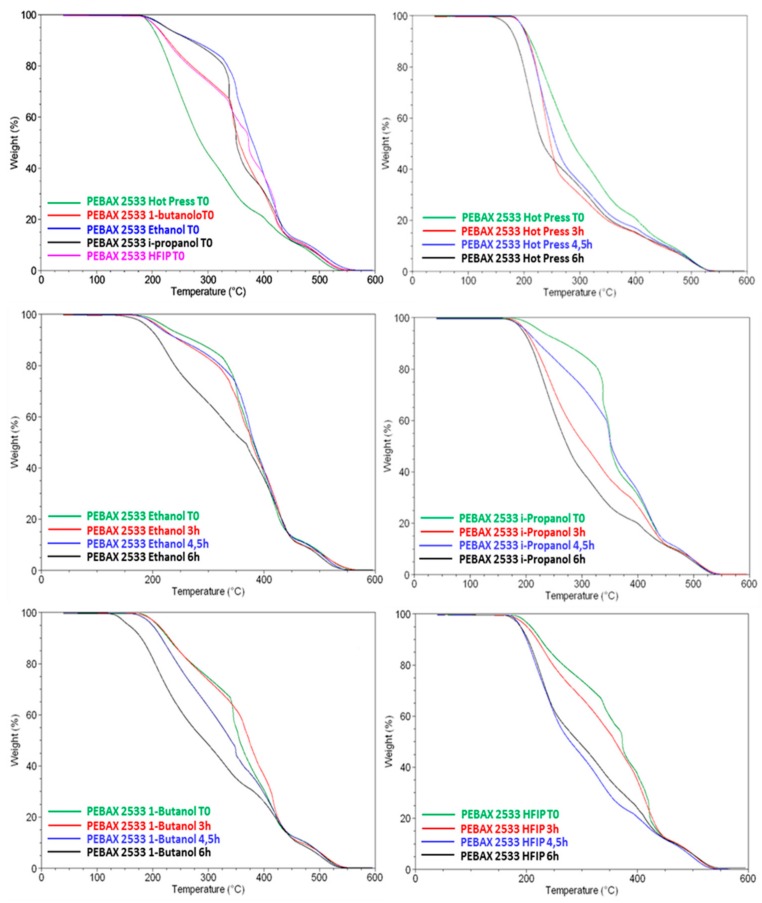
Thermogram overlays from Pebax^®^2533 films at T0 and after 3, 4.5, and 6 h of photo-oxidative degradation.

TGA analyses were performed to determine the presence of residual solvent trapped in the films prepared by solution casting and its eventual influence on permeation parameters. TGA data and thermogram overlays of samples analyzed are reported in Table 4 and in Figure 4, respectively. The films cast from ethanol and 1-butanol showed the presence of residual solvents, with peaks at their correspondent solvent evaporation temperatures. In general, in all films analyzed we observed an initial weight loss from ambient temperature to ca. 240 °C, probably due to absorbed water and the residual solvent trapped in the membranes. The weight loss during this step was within 5%. A second loss stage was observed in the temperature range 300–580 °C and assigned to the thermal decomposition of the main chain scission. Both soft polyether and hard polyamide blocks dramatically decompose in the above mentioned temperature range. Analyzing the weight loss at 50% for the ‘as prepared’ films (T0 samples), the temperature of degradation was higher for films cast from ethanol and HFIP than that of the others (Table 4).

At the different times of photo-exposition, films cast with ethanol display a better thermal stability. Those cast from *i*-propanol, 1-butanol, and HFIP exhibited 50% weight loss at lower temperatures. In particular, at 6 h of photo-exposition, the above mentioned films showed 50% weight loss at temperatures of ca. 270 °C. In the case of solvent-free films, as expected, both temperatures at 5% and 50% weight loss were lower, in the range of 174–199 °C and 230–250 °C, respectively, due to the unavoidable degradation phenomena occurring in the films during the hot press at 200 °C. The residuals at 600 °C (Figure 4) were very similar for all films, confirming the total degradation of the polymer. 

**Figure 5:**
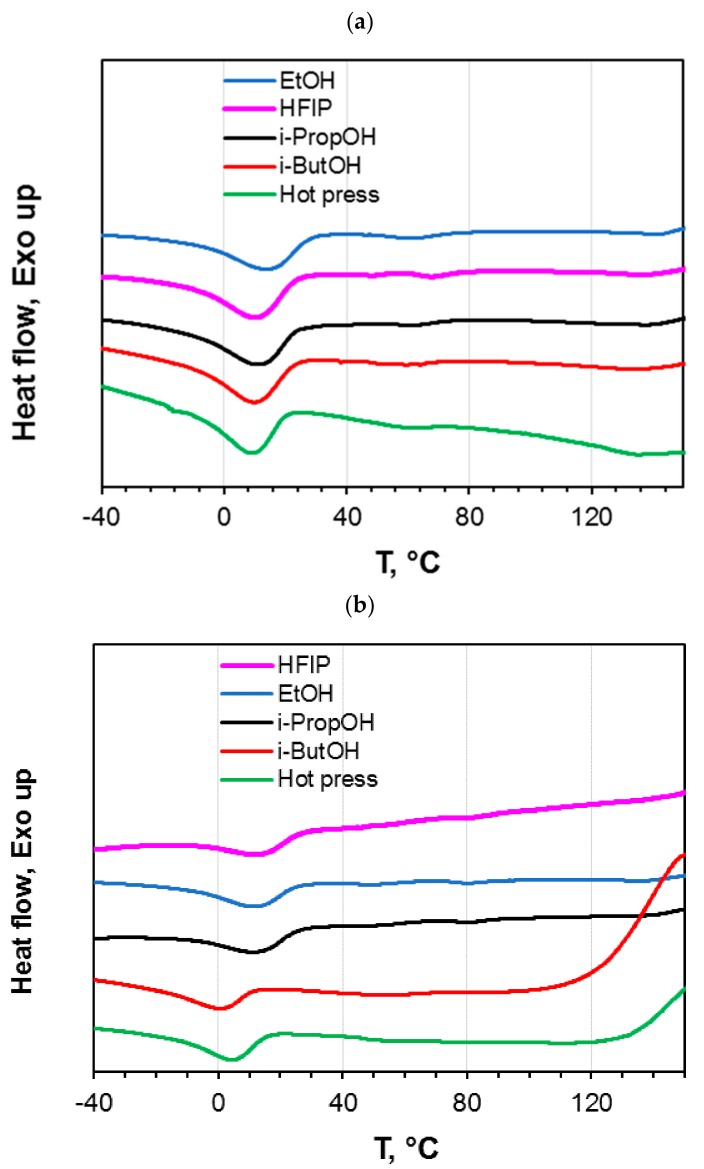
Differential scanning calorimetry (DSC) thermograms (shifted vertically for clarity) relative to the first heating of Pebax^®^2533 films. (**a**) ‘as prepared’ films (T0 samples); (**b**) photo-oxidated samples (6 h).

Structural characteristics of the Pebax^®^ films were investigated by using the DSC analysis. The thermograms obtained during the first heating of untreated and photo-exposed (6 h) samples are shown in Figure 5. The samples display a major melting peak of the PTMO block around 10 °C and a smaller melting peak for the polyamide block around 130 °C. The PA12 melting cannot be clearly observed, particularly in the photo-exposed samples, owing to the small amount of this block in the copolymer. The untreated sample prepared by the hot press method presents a PTMO melting peak at a lower temperature if compared to the other T0 samples (Figure 5a). Indeed, during the cooling from the molten condition associated to the film formation, PA crystallizes first causing a ‘confined crystallization’ for the PTMO phase with a melting point depression. The slight shift observed in the PTMO melting peak for the samples obtained by solution casting reflects roughly the boiling point of the solvent used.

Photo-exposed samples obtained from hot pressing and from 1-butanol casting show the starting of degradation above 130 °C (Figure 4b). This is in agreement with TGA data revealing a low thermo-resistance for such samples. In addition, these two samples present a depression of the melting temperature for the PTMO phase. From the endotherm relative to the melting event of the PTMO sequence, the degree of crystallinity of all samples, either before or after the photo-exposure, was evaluated. Table 5 reports the melting enthalpy of the PTMO block (∆*H*m) for all the samples and the degree of crystallinity (*X*c) estimated by dividing the melting enthalpies by the ∆*H*_0_ of the PTMO block (167 J g^−1^) [20] and considering the PTMO concentration in Pebax (84 wt.%). All samples exposed to photo-oxidation for 6 h show a lower degree of crystallinity than those of untreated samples (T0). This could be due to the chain scission, which is UV induced, that also generates a loss of consistency and thus a diminution of the degree of crystallinity [21]. Samples from ethanol casting display a less pronounced decrease of crystallinity degree upon the photo-oxidation, in agreement with their better thermal stability observed in TGA.

### 3.3. Gas Permeation Properties

The permeation rate tests provided the following transport parameters through the Pebax films: permeability, diffusion, and solubility coefficients for carbon dioxide, methane, oxygen, nitrogen, helium, and hydrogen. In the case of the ‘as prepared’ membranes, the gas transport properties permeability, diffusion, and solubility are summarized in Table 6, Tables S1 and S2, respectively, with the ideal selectivity calculated for some gas pairs.

The permeability data for the ‘as prepared’ Pebax^®^2533 films reveal a role exerted by the preparation method. The solvent-free hot press method produced films having a systematically larger permeability for all gases considered (Table 6). The behavior of the molded films could be ascribed to the inevitable degradation phenomena occurring in the films during hot pressing at 200 °C, as confirmed by the MALDI analysis. However, they had no detrimental impact on the overall gas selectivity. Considering the films prepared by solution casting, some differences can be appreciated depending on the solvent used. The films cast from ethanol or *i*-propanol presented similar permeation properties (Table 6). Close values were found using HFIP as solvent, while films obtained from 1-butanol solutions exhibited larger permeability values. The use of ethanol resulted in a slightly higher permselectivity for different gas pairs.

In the case of block copolymers, the history and thus the solvent used for casting affect their phase–separated microstructures [22] and their crystallinity. Indeed, a different solvent alters the conditions for the polymer dissolution. These changes can be quantified based on the ‘interaction distance’ (∆*δ*), that is the difference of the solubility parameters (*δ*) between the polymer and a solvent [23]:

∆*δ* = [4 ((δ_Dp_ − δ_Ds_)^2^ + (δ_Pp_ − δ_Ps_)^2^ + (δ_Hp_ − δ_Hs_)^2^]^0.5^ (5)

where *δ*_D_, *δ*_P_, and *δ*_H_ are the dispersive (van der Waals), polar (related to the dipole moment), and hydrogen-bonding terms of the solubility parameter, respectively. The subscripts p and s refer to the polymer and solvents, respectively.

Table 7 reports the Hansen solubility parameters (HSPs) for the polymer and the solvents as well as for two gases (i.e., CO_2_ and N_2_). The HSPs listed for Pebax^®^2533 are those reported by Heitmann et al. [24], calculated with the group contribution method. Solvents with lower values of ∆*δ* could dissolve the selected polymer easily. In particular, according to a rule of thumb, solvents with ∆*δ* of lower than eight could be considered suitable solvents for a specific polymer. However, the solubility parameter describes the possibility of dissolving the polymer in a certain solvent, but not the required time and conditions. The individual solubility parameters and the interaction distance reported in Table 7 demonstrate the preferential affinity of the polymer for CO_2_ vs. N_2_, as well as a relatively good interaction between the Pebax and HFIP.

Mohammadi et al. presented a better gas permeability for Pebax^®^1657 films prepared by solvents with increasing molar volume, with the only exception being for dimethylacetamide (DMAc) [25]. This behavior could be related to a ‘template effect’ exerted by the solvent molecules during the membrane formation [26]. In the present study, although the molar volume of HFIP was the largest among the studied alcohols, the permeability of the Pebax^®^2533 film cast from HFIP was lower than that expected by the aforementioned trend. The softer structure in the Pebax^®^2533 was not affected by the solvent ‘template effect’ as could be observed in more rigid structures like in Pebax^®^1657 that has a larger ratio of hard to soft segments [5] or in glassy polymers. Barbi et al., exchanging the casting solvent, confirmed a more significant effect on the domain structure for the Pebax materials having almost 50% hard block content [4]. However, in the case of Pebax^®^2533 films, they measured a reduced permeability when exchanging the solvent from 1-butanol to cyclohexanol. This behavior was explained in terms of an imperfect phase separation with an increased soft domain density due to some hard blocks incorporated in the soft domains.

HFIP is highly polar, strongly hydrogen-bond donating, and very weakly nucleophilic. In the present study, HFIP was the strongest solvent for Pebax^®^2533 among those used (see Table 7), and it produces films with a lower gas permeability. Similarly, DMAc, the strongest solvent for the Pebax^®^1657 in the series used by Mohammadi et al. [25], resulted in membranes with the lowest permeability. A good solvent for the polymer induces a low mobility for the chain segments in the macromolecules; consequently, the polymer chains are more extended so that they form a structure with a higher *T*g [27]. The Pebax^®^2533 copolymer is predominantly constituted by a rubbery semi-crystalline PTMO block. Therefore, the lower permeability found in the film cast from HFIP could be related to the reduced fractional free volume typically observed with increasing *T*g in rubbery amorphous polymers [28].

**Figure 6:**
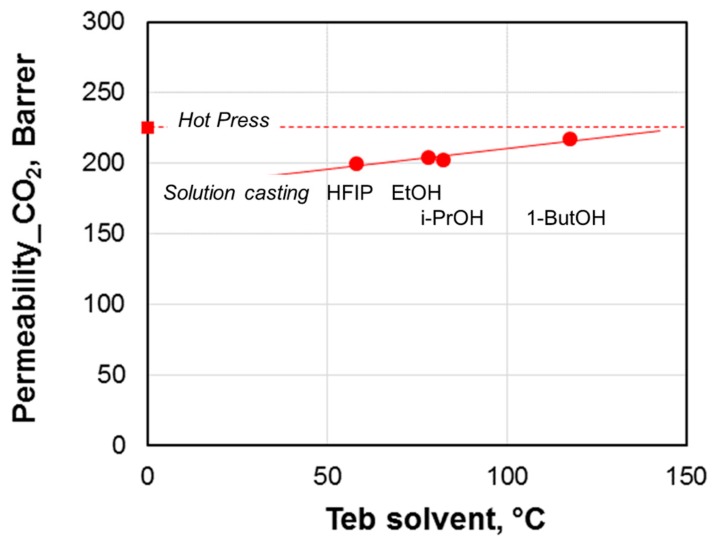
CO_2_ permeability in Pebax^®^2533 films produced from different solvents as a function of the solvent boiling point (T0 samples). Circles: data for the films prepared by solvent evaporation; square: data for the film prepared by the hot press method. Lines are guides for the eye.

In the present work, the differences observed for the CO_2_ permeability in the prepared dense films of similar thickness correlate with the boiling point of the solvent used for casting (Figure 6). Indeed, this property is significantly different between HIFP and 1-butanol. The TGA analyses performed on the virgin Pebax^®^2533 (Figure 4) confirmed the presence of a residual amount of solvent in the case of 1-butanol, which has the highest boiling point. Solvents with a higher boiling point evaporate slowly during the membrane formation, providing adequate time to the polymer chains to facilitate reorganization or increase crystallinity [29,30]. In the present case, DSC effectively evidences a higher crystallinity for the PTMO block in samples cast from 1-butanol. However, this aspect is not predominant on the gas transport properties reported in Table 6, since at room temperature the melting of PTMO is almost complete (Figure 5). Therefore, the larger permeability found in the film cast from 1-butanol could be related to a swelling dilation induced by the solvent since in rubbery polymers penetrants with a higher boiling point are more sorbed, leading to an enhanced plasticization of the polymer. On the other hand, HFIP, having the lowest boiling point (see Table 1), has no residue in the cast films. Therefore, as discussed before, the affinity of HFIP with the Pebax^®^2533 and the absence of residual solvent have a relevant role in determining the copolymer structure and thus the gas permeation properties.

According to the rubbery state of the PTMO block, in all tested films the permeation order for the different gases does not follow the penetrant molecular size as found in glassy materials such as polycarbonates, polyimides, and polypyrrolones [31]. CO_2_ was the most permeable species among the gases considered. Indeed, the presence of polar groups into the polymer matrix, due to the polyethylene oxide, enhances the solubility selectivity of CO_2_ over light gases, as proved by the good affinity evaluated on the basis of the Hansen solubility parameters (Table 7). CO_2_, having a quadrupole moment, interacts favorably with such polar groups [32]. In rubbery polymers, the CO_2_ solubility is higher than that of other permanent gases, but the polymer chains mobility results in a reduced ability to sieve penetrant molecules on the basis of their molecular size. Therefore, only moderate changes in gas selectivity can be appreciated by switching to a different casting solvent.

The smaller gases, such as H_2_ and He, are the more mobile in all the prepared membranes as suggested by their large diffusion coefficient (Table S1). Instead, the solubility of CO_2_ is the highest in all samples (Table S2) and its contribution results in the higher permeability of CO_2_ with respect to other gases. Therefore, all the samples prepared are ‘reverse selective membranes’ that display a preferential separation for larger molecules that are more sorbed than smaller molecules [33]. Films cast from HFIP present a general decrease of diffusion and increase of solubility coefficients for the different gases tested (Tables S1 and S2). However, the reduced permeability depends on a predominant role of diffusion.

#### 3.3.1. Effect of Temperature on Gas Permeation

Due to the polar nature of this membrane material, Pebax^®^2533 has been investigated for separating polar and non-polar gas pairs. An example is the CO_2_/N_2_ separation that is temperature sensitive with Pebax^®^2533; the predominant solubility contribution for CO_2_ is depressed by increasing the temperature and thus a higher selectivity is achieved at lower temperature.

The gas permeability of all Pebax films increased with temperature, indicating a diffusion–dominated gas transport mechanism in the temperature range tested. The temperature dependency of permeation parameters can be approximated by an Arrhenius expression:

*P* = *P*_0_*exp*(−*Ep*/*RT*) (6)

where *P*_0_ is a pre-exponential factor and *Ep* is the apparent activation energy for permeation [34].

In all tested films, the order of *Ep* was N_2_ > CH_4_ > O_2_ > H_2_ ≈ He > CO_2_ (Table 8), indicating the greatest influence of temperature on the less permeable species (e.g., N_2_). Accordingly, the permselectivity is generally depressed by increasing the temperature because the less permeable species present a higher activation energy than more permeable molecules.

The activation energy of gas permeation is similar in the films prepared from ethanol, *i*-propanol, 1-butanol, or by the hot press method (Table 8). Larger *Ep* values were evaluated for the films obtained from HFIP, indicating a more pronounced sensitivity with respect to temperature changes, but also a denser structure. Typically, polymers with a higher fractional free volume (FFV) present a lower activation energy for the transport of gases [31]. Indeed, a large FFV facilitates the permeation to gas molecules with less resistance, resulting in a simultaneous decrease in the activation energies for permeation [31]. The obtained results indicate small differences in the FFV.

#### 3.3.2. Effect of Photo-Oxidation on Gas Permeation

**Figure 7:**
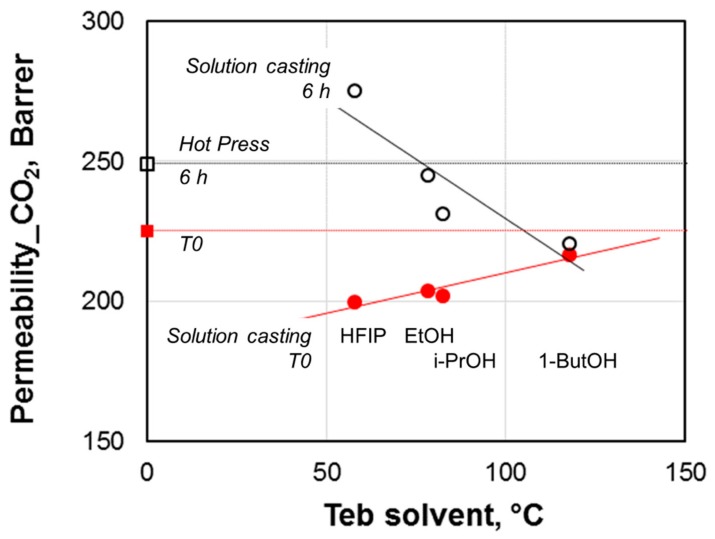
CO_2_ permeability in Pebax^®^2533 films produced from different solvents as a function of the solvent boiling point. Circles: data for the films prepared by solvent evaporation; squares: data for the films prepared by the hot press method. Filled symbols: data for ‘as prepared’ (T0) films; open symbols: data for photo-oxidated films (6 h).

The Pebax^®^ films were also defect-free after their photo-oxidation for irradiation times up to 6 h. The films tested after the 6 h of photo-oxidative stress presented a larger permeability than the ‘as prepared’ films (Figure 7). The increment in permeability is also evidenced for the film obtained by the hot press method. The films cast from HFIP and ethanol showed larger gains in gas permeability after the photo-oxidative treatment. The gain in permeability could be related to a removal of some residual solvent and, thus, it is more efficient for the solvents with a lower boiling point. TGA of photo-exposed Pebax^®^ samples did not exhibit the presence of entrapped solvent. Actually, the UV irradiation at 60 °C, in presence of fan air for prolonged time, allows for solvent evaporation during the photo-oxidation process. In the case of 1-butanol, the permeability is not much affected by the photo-oxidation because the effect of the solvent–induced dilation is removed.

The chemical changes produced by the photo-oxidation can result in a reduced effective thickness for the dense films. Indeed, the photo-oxidative degradation causes damage up to a few microns below the surface [35]. Undeniably, in the case of the films prepared from ethanol and HFIP, their surfaces were much affected by the UV treatment as evidenced by the contact angle measurements (Table 3). According to a resistance in series model [36], considering the photo exposed film as a bi-layer structure with a very permeable oxidized layer and no altered bulk, the thickness of the modified layer can be estimated. This evaluation can be done using the permeability of each gas, and an average value can be obtained for six gases. The films cast in 1-butanol seem unaffected by the photo-oxidation, whereas the modified thickness increases up to 30% of the total for the films cast from HFIP. In the case of the molded films, this layer is less than 10% of the total.

## 4. Discussion

The effect of various casting solvents on the microstructure and gas-permeation performance of dense Pebax^®^2533 membranes, obtained via the solution casting method followed by controlled solvent evaporation, is demonstrated. The gas transport properties reveal a dependence of permeability on the boiling point of the solvent used, but no significant effect on the membrane selectivity. Among the alcohols used for casting, the HFIP fluoroalcohol produces the less permeable samples, while ethanol results in slightly more selective films. In the case of HFIP, which is highly polar, the large affinity of this solvent with the copolymer affects the microstructure and, thus, the permeation parameters.

The solvent-free molding process results in more permeable films, owing to some degradation phenomena induced during the film formation.

The gas transport is consistent with the data collected by thermal and spectrometric analyses. TGA analyses give information on the presence of residual solvent entrapped into the membranes, showing the presence of the 1-butanol that results in a swelling effect. MALDI MS reveals a higher content of hydrophilic chain ends on the molded samples and, in general, on the photo-exposed films. A controlled photo-oxidation treatment (up to 6 h) is effective in increasing the gas permeability of the Pebax^®^ films, keeping their selectivity, whereas the films treated for a period of 11 h became fragile and could not be tested. The photo-oxidation process results in a reduction of the crystallinity of the Pebax^®^2533 films, as found by DSC, as well as of the hydrophobicity, as shown by water contact angle measurements and supported by MALDI analyses. A better thermal resistance is shown by TGA analysis for the films prepared in ethanol that also present the larger permeability gain upon photo-oxidation, keeping a hydrophobic character.

Pebax^®^2533, as already reported with the addition of ionic liquids [5], can be modified to a lesser extent than the more rigid grades (e.g., Pebax^®^1657). Indeed, the more rigid segments of the copolymers could be plasticized by suited additives, but in Pebax^®^2533 they are the minority blocks. However, the surface properties can be tuned by an accelerated aging induced by the photo-oxidation, without compromising the selectivity. Typically, hydrophilic membranes have a lower membrane fouling potential than hydrophobic ones because proteins and many other foulants are hydrophobic in nature [37]. Therefore, many attempts have been made to increase the membrane surface hydrophilicity by surface modification [38].

Despite the higher solvent power of HFIP, ethanol, being also a green solvent, can be considered the best choice to process the Pebax^®^2533 copolymer in order to obtain membranes with a dense layer for application in gas separation or pervaporation. The information provided by this study can be used for the development of thin film composite membranes or mixed matrix membranes, whereas data related to the molded films obtained by the hot press procedure could be useful for preparing thicker dense films (e.g., for food packaging applications).

## Figures and Tables

**Table 1:** Properties of the polar solvents used in this study. HFIP = hexafluoroisopropanol.

Solvent	Molar Volume (mL mol^−1^)	Density (g mL^−1^)	Boiling Point (°C)
ethanol	58.5	0.789	78.2
*i*-propanol	76.8	0.803	82.3
1-butanol	91.5	0.810	117.7
HFIP	105.3	1.60	58.2

**Table 2:** Oligomer structures assigned to the mass ions appearing in the matrix-assisted laser desorption ionization (MALDI) spectra of photo-oxidized and pure Pebax^®^2533 samples.

Structures	M·H^+^	M·Na^+^	M·K^+^
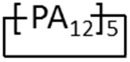	-	1009.13	-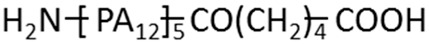	-	1154.25	-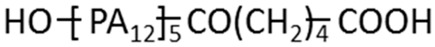	1133.14	1155.23	1171.18
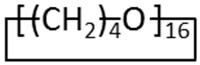	-	1176.17	-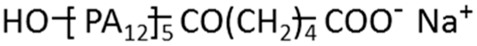	-	1177.20	-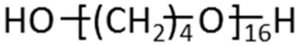	-	1194.20	-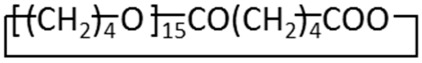	-	1232.21	-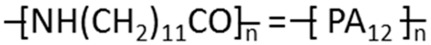			

**Table 3:** Water contact angle measured on the ‘as prepared’ and photo-oxidated Pebax^®^2533 films.

Preparation Method	Casting Solvent	Water Contact Angle (°)	
		T0	6 h
Solution casting	ethanol	112.49 ± 0.34	94.11 ± 0.70
	*i*-propanol	87.89 ± 0.33	87.88 ± 1.18
	HFIP	88.91 ± 1.31	83.06 ± 0.50
	1-butanol	94.40 ± 0.90	93.65 ± 0.60
Hot press	-	83.77 ± 0.51	82.72 ± 1.11

**Table 4:** Thermogravimetric analysis (TGA) properties of Pebax^®^2533 films at T0 and after 3, 4.5, and 6 h of photo-oxidative degradation.

Exposure Time (h)	T (Δm = 5%) ^a^ (°C)	T (Δm = 50%) ^b^ (°C)	R^c^ (%)
PEBAX 2533 ethanol			
**T0**	224.9	379.7	0.15
**3**	213.4	377.4	0.10
**4.5**	210.3	381.2	0.10
**6**	192.1	362.9	0.11
**PEBAX 2533 *i*-propanol**			
**T0**	224.1	350.7	0.01
**3**	198.3	289.5	0.13
**4.5**	200.2	353.0	0.04
**6**	193.1	274.0	0.01
**PEBAX 2533 1-butanol**			
**T0**	208.4	356.1	0.15
**3**	210.2	372.8	0.17
**4.5**	199.7	340.0	0.17
**6**	156.8	272.9	0.10
**PEBAX 2533 HFIP**			
**T0**	208.4	372.8	0.01
**3**	200.5	359.9	0.01
**4.5**	189.0	278.5	0.11
**6**	189.2	293.5	0.48
**PEBAX 2533 Hot Press**			
**T0**	199.3	286,9	0.10
**3**	199.2	247.6	0.22
**4.5**	198.1	256.1	0.01
**6**	174.5	233.3	0.12

^a^ Onset temperature for decomposition (5% loss of initial weight). ^b^ Decomposition temperature at 50% loss of initial weight. ^c^ Weight residue (%) at 600 °C.

**Table 5:** Thermal data of the poly (tetramethylene oxide) (PTMO) melting peak from DSC curves for ‘as prepared’ films (T0 samples) and photo-exposed Pebax^®^2533 films (6 h).

Preparation Method	Casting Solvent	∆*H*m (J g^−1^)		*X*c (wt.%)	
		T0	6 h	T0	6 h
Solution casting	ethanol	32.8	31.2	23.4	22.3
	*i*-propanol	38.3	30.1	27.3	21.4
	HFIP	35.8	22.5	25.5	16.0
	1-butanol	47.0	30.2	33.5	21.5
Hot press	-	36.2	29.4	25.8	21.0

**Table 6:** Gas permeability of Pebax^®^2533 at 25 °C. ‘As prepared’ films (T0 samples).

Preparation Method	Solvent	Permeability (Barrer)						Selectivity (–)		
		CO_2_	CH_4_	O_2_	N_2_	He	H_2_	CO_2_/N_2_	H_2_/N_2_	O_2_/N_2_
Solution casting	ethanol	204	25.6	19.7	7.4	21.2	37.4	27.3	5.03	2.65
	*i*-propanol	202	25.4	19.3	7.8	21.7	37.9	25.8	4.84	2.47
	1-butanol	217	26.8	21.8	9.1	25.4	43.1	23.9	4.75	2.41
	HFIP	200	25.2	20.3	8.4	24.0	38.5	23.9	4.61	2.40
Hot press	-	225	28.8	22.7	8.9	25.1	43.7	25.3	4.91	2.55

^1^ Barrer = 10^−10^ cm^3^ (STP) cm cm^−2^ cmHg^−1^ s^−1^. STP: standard temperature and pressure (0 °C and 1 atm).

**Table 7:** Hansen solubility parameters (HSPs) of Pebax^®^2533, some polar solvents, and some gases.

	Solubility Parameter (MPa)^0.5^					
Material	*δ*_D_	*δ*_P_	*δ*_H_	*δ*_t_^a^	∆*δ*	Ref.
Pebax^®^2533	17.6	7.6	6.8	20.3	-	[24]
HFIP	17.2	4.3	14.7	23.0	8.6	[23]
*i*-Propanol	15.8	6.1	16.4	23.6	10.4	[23]
1-Butanol	16.0	5.7	15.8	23.2	9.7	[23]
Ethanol	15.8	8.8	19.4	26.5	13.2	[23]
Water	15.5	16.0	42.3	47.8	43.3	[25]
CO_2_	15.7	6.3	5.7	17.9	4.2	[25]
N_2_	11.9	0	0	11.9	15.3	[25]

^a^*δ*_t_, total cohesion (solubility) parameter: *δ*_t_^2^ = *δ*_D_^2^ + *δ*_P_^2^ + *δ*_H_^2^.

**Table 8:** Apparent activation energy for permeation (*Ep*), evaluated in the temperature range 15–55 °C, for Pebax^®^2533 films (‘as prepared’).

Preparation Method	Solvent	Activation Energy for Permeability (kJ mol^−1^)					
		CO_2_	CH_4_	O_2_	N_2_	He	H_2_
Solution casting	ethanol	18.5	30.8	29.5	34.4	28.0	27.6
	*i*-propanol	18.6	31.0	30.1	34.6	28.3	28.0
	1-butanol	18.4	30.8	29.3	33.2	26.2	28.3
	HFIP	21.9	34.5	31.9	37.9	31.9	30.7
Hot press	–	19.0	31.2	30.3	34.5	28.2	28.1

## Supplementary Materials

The following are available online at http://www.mdpi.com/1996-1944/11/8/1326/s1, Table S1: Diffusion coefficients of Pebax®2533 films at 25 °C (T0 samples), Table S2: Solubility coefficients of Pebax^®^2533 films at 25 °C (T0 samples).
