# Supplementary material for: Influence of the Preparation Method and Photo-Oxidation Treatment on the Thermal and Gas Transport Properties of Dense Films Based on a Poly(ether-block-amide) Copolymer
Source: Materials (Basel). 2018 Jul 31;11(8):1326. doi: 10.3390/ma11081326 (PMC6119977; doi:10.3390/ma11081326)
Supplement: Supplementary file 1 [file materials-11-01326-s001.pdf]

Supplementary Information

Table S1. Diffusion coefficients of Pebax®2533 films at 25 °C (T0 samples).

Solvent	Diffusion coefficient (10^{-8} cm^2 s^{-1})						Selectivity (–)	
	**CO_2**	**CH_4**	**O_2**	**N_2**	**He**	**H_2**	**CO_2/N_2**	**H_2/N_2**
ethanol	133	106	204	170	2300	1020	0.78	6.0
i-propanol	135	102	220	202	1880	1070	0.67	5.3
1-butanol	133	124	221	153	2590	1110	0.87	7.2
HFIP	124	99.5	203	135	2620	1110	0.92	8.2
Hot press	158	121	270	201	3030	1390	0.79	6.9

Table S2. Solubility coefficients of Pebax®2533 films at 25 °C (T0 samples).

Solvent	Solubility coefficient (cm^3 cm^{-3} bar^{-1})						Selectivity (–)	
	**CO_2**	**CH_4**	**O_2**	**N_2**	**He**	**H_2**	**CO_2/N_2**	**H_2/N_2**
ethanol	1.15	0.18	0.072	0.033	0.0069	0.027	35.0	0.84
i-propanol	1.12	0.19	0.066	0.029	0.0087	0.027	38.7	0.92
1-butanol	1.22	0.16	0.074	0.041	0.0074	0.029	27.4	0.66
HFIP	1.21	0.19	0.075	0.046	0.0069	0.026	26.1	0.56
Hot press	1.07	0.18	0.063	0.033	0.0059	0.022	32.2	0.71
